# Cost estimation of the National Essential Public Health Service Package in township health centers in Guangxi, China: based on the equivalent method

**DOI:** 10.1186/s12889-025-24347-y

**Published:** 2025-09-30

**Authors:** Shuyun Wang, Yilin Zhao, Xia Liang, Jing Kang, Yujun Chen, Huihan Zhao, Lin Lin, Ying Zhang, Zhaoquan Huang, Qiming Feng

**Affiliations:** 1 Health and Policy Research Center, Guangxi Medical University, Nanning, 530021 Guangxi People’s Republic of China; 2 College of Business and Economics, Australian National University, Canberra, ACT 2601 Australia; 3 Guangxi Medical University, Nanning, 530021 Guangxi People’s Republic of China; 4 Department of Hematology, The First Affiliated Hospital of Guangxi Medical University, Nanning, 530021 Guangxi People’s Republic of China; 5 The First Affiliated Hospital of Guangxi Medical University, Nanning, 530021 Guangxi People’s Republic of China

**Keywords:** National public health service package, Cost estimation, Equivalent method, Service efficiency

## Abstract

**Background:**

Since the 2009 healthcare system reform, China’s National Essential Public Health Service Package (NEPHSP) has made notable progress. However, due to low levels of informatization and limited attention to rural areas, existing research has primarily focused on urban settings, with few studies examining cost estimation and its practical application in rural regions. Moreover, cost estimation findings are rarely used to inform grassroots management practices. This study aims to provide empirical support for improving the efficiency and quality of the NEPHSP at the grassroots level.

**Methods:**

This study employed a multi-stage stratified sampling method to randomly select 50 township health centers in Guangxi. Data on revenue, expenditure, and workload related to primary medical services and the NEPHSP were collected. The equivalent method was used to estimate service costs, and correlation analysis and analysis of variance were conducted to identify factors influencing costs and workload.

**Results:**

Per capita actual subsidy (CNY 57.78) < per capita actual expenditure (CNY 60.35) < per capita allocated subsidy (CNY 74) < per capita estimated cost (CNY 110.96). There was a weak positive correlation between the actual revenue from the NEPHSP and county-level GDP (*r* = 0.375, *P* < 0.05), and a weak positive correlation between the per capita estimated cost and the proportion of total equivalent value of the NEPHSP (*r* = 0.414, *P* < 0.01). Analysis of variance indicated significant differences in the equivalent value proportions of 13 NEPHSP categories and in the unit equivalent values for some services across counties (*P* < 0.05).

**Conclusion:**

This study is expected to generate key evidence on the relationships and influencing factors among different per capita costs, as well as the workload and unit efficiency of the NEPHSP, providing empirical support for optimizing resource allocation and improving the efficiency and quality of the NEPHSP.

## Introduction

Since China’s healthcare system reform in 2009, the equalization of the National Essential Public Health Service Package (NEPHSP) has achieved positive results. First, an essential public health service system has been established, with three types of primary healthcare institutions—community health service centers, township health centers and village health offices, providing the National Essential Public Health Services free of charge to registered residents under their jurisdictions. Second, the level of equalization of public health services has been gradually improved, with the content of services increasing from 10 major categories initially to 12 major categories [1, 2], and owing to a stable and increasing funding mechanism, per capita allocated subsidies rose from CNY 15 in 2009 to CNY 74/person in 2020 [3–5]. Third, the coverage rate of all types of services has increased substantially, the issuance rate of vaccination certificates and vaccination coverage have both remained above 90% [6], while the establishment and utilization rates of health records increased from 48.78% to 22.14% in 2009 to 88.25% and 55.34% in 2019, respectively, indicating significant effectiveness in health management [7].

With continuous annual increase in subsidy funds, the NEPHSP budget target reached CNY 61,605,330,000 in 2023, of which CNY 3,031,870,000 (4.92%) was allocated to Guangxi Zhuang Autonomous Region (Guangxi) [8]. As the NEPHSP are government-funded public goods serving the public interest, and their performance is supervised by administrative departments, scientific cost estimation has become an essential part of improving the project compensation mechanism. Over the past decade, Numerous studies have explored and practiced the estimation of the NEPHSP, leading to the development of three main methods: First, the equivalent method, which evolved from the Workload Indicators of Staffing Need (WISN) method that proposed by the World Health Organization (WHO) for estimating staffing needs of health workers [9]. In 2015, Bowen and Delu applied the equivalent method to develop a scientifically sound and simple cost estimation framework for community health service projects in China. This method can flexibly adapt to changes in the scope of service items and is suitable for environments with low levels of information technology and high population density at the grassroots level [10–12]. Second, the full cost method, a representative of top-down estimation approaches. This method is relatively coarse and depends on weighting and allocation coefficients, necessitating a high degree of standardization in grass-roots implementation [13–15]. Third, the activity-based costing method, a representative of bottom-up estimation approaches. It involves breaking down the entire service package into multiple operational steps and conducting step-by-step cost estimation. This method requires a high level of data accuracy and is suitable for organizations with multiple layers, complex structures, and detailed activity records [11, 16–18]. Considering that township health centers in rural areas are characterized by low levels of informatization, and that labor costs and efficiency are key research considerations, the equivalent method was selected for this cost estimation (see Table 1).

**Table 1:** Comparison of three costing methods

Method	Applicable Scenario	Advantages	Limitations
Equivalent Method	Suitable for institutions with low levels of informatization.	Can quickly estimate costs; focuses on labor costs, emphasizes work volume comparison; plays an irreplaceable role in new projects.	Considers only labor costs, neglects indirect costs, affecting the accuracy of cost estimation.
Full Cost Method	Suitable for regional budget allocation.	Emphasizes the overall picture, advantageous for total and average costs.	If involving multiple institutions and levels, cost precision is relatively poor; significantly influenced by weights and allocation coefficients.
Activity-Based Cost (ABC)	Suitable for institutions with high levels of informatization.	Emphasizes individual services, helps accurately determine the cost composition of each service, optimizes resource allocation.	Requires a high level of information systems, high data collection costs, complex calculation processes.

Township health centers are the central link in China’s three-tier rural preventive healthcare network which consists of county hospitals, township health centers, and village clinics. Their main function is to safeguard the health of local residents by providing primary medical services and the NEPHSP. By the end of 2020, there were 35,762 township health centers across 30,000 townships in China, recording a total of 1.1 billion outpatient visits, far exceeding the 130 million visits reported by community health service centers [19, 20]. However, existing studies have taken into account the level of improvement in institutional information systems and data availability, with most NEPHSP cost estimation studies conducted in urban communities [11, 16, 21, 22], and relatively fewer studies focusing on township health centers. Meanwhile, since the implementation of the NEPHSP, financial input has increased year by year with an average annual increase of about CNY 5 per person, however, a decade, the sustainability of this funding mechanism remains limited [23, 24]. Most of the existing studies recommend strengthening cost management, increasing financial input and establishing a diversified financing system to address funding shortages [24–26]. Few studies have used cost estimation results to guide grassroots managers. Our study seeks to address this gap by exploring how these results can inform strategies to improve efficiency and quality of medical staff services under limited financial resources, ultimately promoting more effective basic public health service equalization.

Situated in southern China and bordered by the sea of the Beibu Gulf in the south, Guangxi is one of the five major ethnic minority autonomous regions in China, with 14 cities, 12 hereditary ethnic groups (11 of which are ethnic minorities), and a registered residents of 50,126,800 people. Guangxi is a less derdeveloped region in China, with a GDP of CNY 2215.669 billion and a per capita disposable income of CNY 24,562 (€ 3,121.58/$ 3,559.86) in 2020. A total of 1,265 township health centers in Guangxi provide the NEPHSP free of charge to residents. These centers are government-funded and supervised by the health administrative authorities. Guangxi adopts grid-based organizational management to implement the NEPHSP. At the district level, tasks are assigned annually in line with the nationally updates; the county level refines relevant policies as directed by the district, and the township health centers are responsible for the concrete implementation. Between 2010 and 2020, the average life expectancy in Guangxi increased from 75.10 to 77.52 years, the maternal mortality rate declined from 18.88 to 8.37 per 100,000, and the mortality rate of children under 5 years old has decreased from 7.65 per thousand to 3.77 per thousand, so the implementation of the project has played a positive role in the promotion of residents’ health in the whole region. The Guangxi government has prioritized standardized management of special funds for people’s livelihoods. In terms of fund allocation, the cost-sharing ratio of all levels of finance is set at 80%from the central government, 12% from the autonomous region, and 8% from cities and counties. Since 2012, performance-based budget management has been applied, with third-party audits ensuring effective used of these funds, so as to ensure the utilization rate of the special funds for people’s livelihood.

Therefore, our primary objective was to use the equivalent method to measure the cost of the basic public health service in township health centers in Guangxi and to explore the match between the financial inputs, workloads and quality of work in township health centers from the perspective of financial management of the project, with a view to completing the practical test of the equivalent method in township health centers, providing the basis and suggestions for improving the compensation mechanism of the project and enhancing the efficiency and quality of the service of the medical staff.

## Methods

### Sampling

According to the government’s Five-Zone Economic Development Plan, Guangxi is divided into five regions: Gui-dong (Eastern), Gui-nan (Southern), Gui-xi (Western), Gui-bei (Northern), and Gui-zhong (Central). In consideration of the overall scale of each region, a simple random sampling method was applied in the first stage. Specifically, one city was randomly selected from each economic zone, forming the first-stage sample of five cities.

Given that the administrative structure of Guangxi consists of three hierarchical levels—cities, counties, and townships, to ensure both research feasibility and sample representativeness, two counties were randomly selected from each of the five sampled cities in the second stage, yielding a second-stage sample of 10 counties.

In the third stage, five townships were randomly selected from each of the 10 selected counties, resulting in a total of 50 townships. All township health centers within each selected township were included as survey units, thus yielding a final sample of 50 township health centers.

### Equivalent method for measuring the costs of the NEPHSP

#### Determining costs

In this study, the operational cost of township health centers in Guangxi includes the cost of primary medical services and the NEPHSP. To ensure the accuracy and reliability of the cost data, financial information from 50 sampled institutions was extracted uniformly from the 《2020 Annual Expenditure Statement》 in the financial annual report.

#### Determining the categories and sub-services of Township health centers

Based on relevant studies conducted in China [27, 28], an initial questionnaire was developed to assess workload. This questionnaire was further refined based on a pilot survey. Given the functional orientation of township health centers, their operational activities are categorized into two main types: primary medical services and the NEPHSP. Therefore, the workload questionnaire covers both categories.

Primary medical services are divided into five categories encompassing 71 specific items: clinical medical services, nursing services, pharmaceutical services, medical technology services, and public health services (distinct from the NEPHSP and referring mainly to public health support services associated with clinical diagnosis and treatment). The NEPHSP include thirteen categories with a total of 37 items: establishment of resident health records health education, preventive inoculation, children health management, maternal health management, elderly health management, chronic disease management (hypertension), chronic disease management (type 2 diabetes), severe mental disorder management, tuberculosis patient management, traditional Chinese medicine health management, reporting and management of infectious diseases and public health emergencies, and Supervision and co-management of health and family planning.

The survey collected data on the average number of health workers and time (minutes) required per service instance, the annual quantity of each service item, and the number of registered residents covered by the services.

#### Steps for measuring costs by the equivalence method

**Fig. 1:**
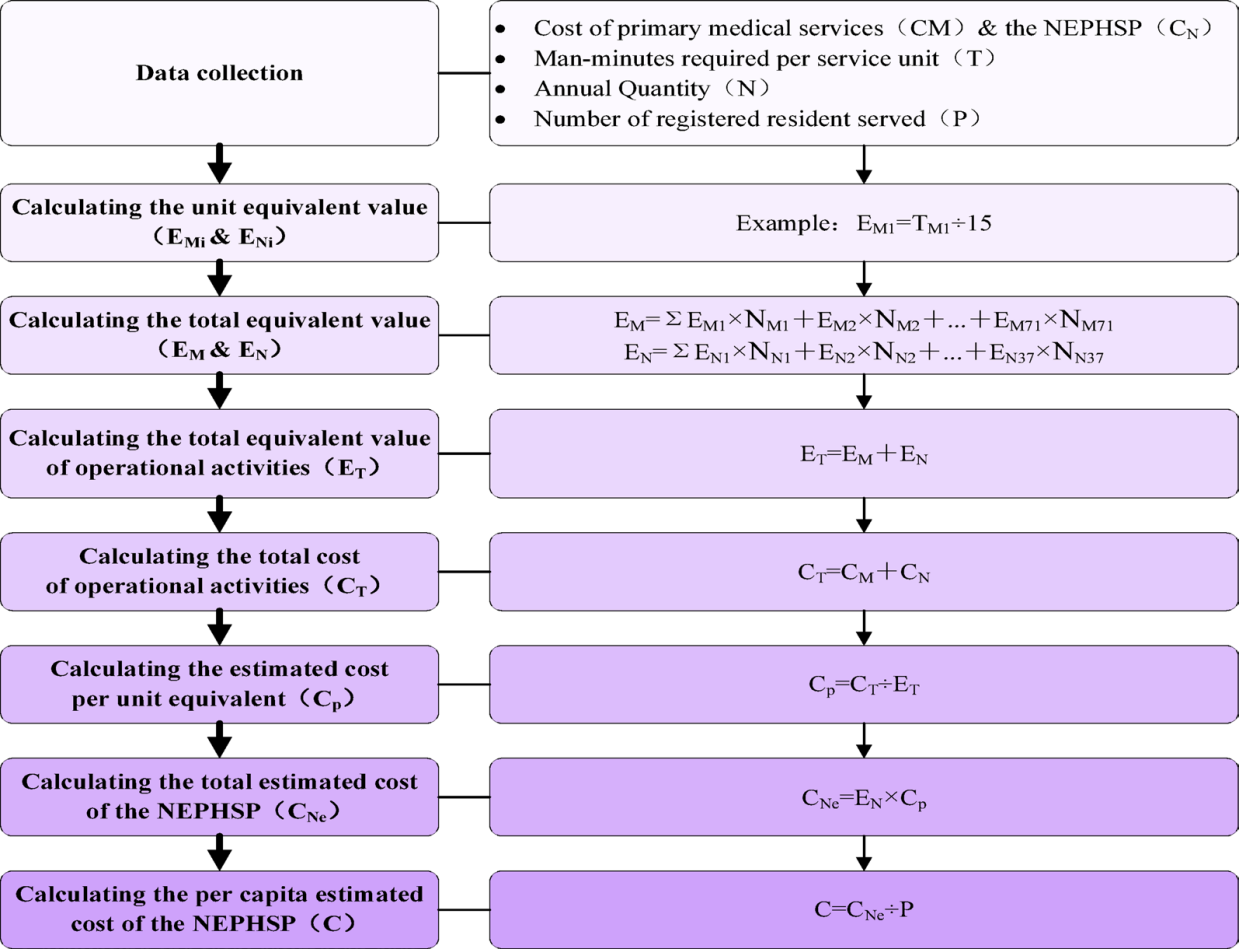
Steps for Calculating the Costs of the NEPHSP Using the Equivalent Method

The equivalence method known for its reliability and good applicability at the grassroots level, enables standardization of service volumn across different types [29]. Fowlling a cost estimation model developed by Yin based on the equivalence method and the NEPHSP in China, one standard service equivalent is defined as “the workload of a health worker seeing one patient during a 15-minute consultation” [27]. This serves as the benchmark for other calculation. For example, if it two health workers collaborate for 12 min on a vision screening service for one child at township health center A, then the man-minutes required per service unit is 24 person-minutes (2 people × 12 min), yielding an equivalent value of 1.6 (24 person-minutes ÷ 15 person-minutes). The steps for measuring the costs of essential public health service programs using the equivalence method are as follows: (Fig. 1)

### Statistical method

Data were entered and organized using WPS 2025, and descriptive statistical analysis was performed using SPSSAU.

Spearman’s rank correlation coefficient was employed to assess the correlations between: (1) NEPHSP revenue of sampled institutions and the county-level gross domestic product (GDP); (2) per capita estimated cost, the number of registered residents served, and the total NEPHSP equivalent value; (3) the total equivalent value, unit equivalent value, and annual service cases.

Analysis of variance (ANOVA) was performed to compare total equivalent values among NEPHSP categories, and to exame differences in unit equivalent values for the same category across different counties.

## Results

### Demand and supply of health services and institutional finances in sample Township health centers

In 2020, the 50 sampled institutions serves a total of 1,695,105 resident, and employed 2,199 health professionals. On average, each institution served 33,902 registered residents and employed 44 health professionals. Among the five regions, the eastern region had the largest registered resident population served and highest number of health professionals per institution, while the western region had the lowest for both measures (see Table 2).

The total actual revenue from the NEPHSP for the sampled institutions was CNY 97,939,575.40, accounting for 20.76% of their total institutional operating income, with an average actual subsidy of CNY 57.78 per capita. Spearman’s correlation analysis indicated a weak positive correlation between the actual revenue from basic public health service projects and the county-level GDP (*r* = 0.375, *P* < 0.05).The total actual expenditure on these projects was CNY 102,293,282.85, representing 16.47% of the total institutional operating expenditure, with an average actual expenditure of CNY 60.35 per capita (see Table 3).

**Table 2:** Status of institutions and personnel in sample regions

Region	Number of township health centers (Institutions)	Number of registered residents served (People)		Number of Health Care Professionals (People)	
		Total	Mean ± SD	Total	Mean ± SD
Eastern	10	586,297	58,630 ± 23,051	632	63 ± 21
Southern	10	364,804	36,480 ± 32,309	439	44 ± 28
Western	10	180,562	18,056 ± 6,801	328	33 ± 10
Northern	10	301,082	30,108 ± 33,877	350	35 ± 18
Central	10	262,360	26,236 ± 10,185	450	45 ± 12
Guangxi	50	1,695,105	33,902 ± 26,832	2199	44 ± 21

**Table 3:** Actual income and expenditure of the NEPHSP in sample regions

Region	Revenue				Expenditure			
	Total	%	Mean ± SD	Per Capita	Total	%	Mean ± SD	Per Capita
Eastern	39,531,791.00	16.35	3,953,179.1 ± 1,484,254.33	67.43	37,626,954.63	19.81	3,762,695.46 ± 1,412,772.94	64.18
Southern	14,657,212.10	19.44	1,465,721.21 ± 848,201.13	40.18	16,095,246.19	21.63	1,609,524.62 ± 681,663.33	44.12
Western	11,897,644.58	14.21	1,189,764.46 ± 474,328.60	65.89	12,233,751.26	17.83	1,223,375.13 ± 489,935.09	67.75
Northern	13,589,591.18	14.84	1,358,959.12 ± 1,126,892.16	45.14	18,494,088.76	26.42	1,849,408.88 ± 1,999,417.75	61.43
Central	18,263,336.54	17.87	1,826,333.65 ± 646,476.39	69.61	17,843,242.01	19.85	1,784,324.20 ± 731,279.43	68.01
Guangxi	97,939,575.40	16.47	1,958,791.51 ± 1,395,413.92	57.78	102,293,282.85	20.76	2,045,865.66 ± 1,459,042.92	60.35

### Cost Estimation of the NEPHSP

The per capita estimated cost of the NEPHSP across the 50 township health centers was CNY 110.96, with the central region reporting the highest cost(CNY 128.49) and the southern region reported the lowest was in (CNY 69.59). The NEPHSP activities accounted for 38.16% of the total equivalent value of the institutional operational operation, ranging from a maximum of 50.70% in the northern region to a minimum of 34.10% in the southern region. The cost per standard service equivalent was CNY 18.00, reflecting the human resource expense for a health professional to complete 15-minutes service workload (see Table 4).

Spearman’s correlation analysis illustrated a weak negative correlation between the per capita estimated cost and the number of registered residents served (*r* = − 0.312, *P* < 0.05), and a weak positive correlation between the per capita estimated cost and the proportion of total equivalent value of the NEPHSP (*r* = 0.414, *P* < 0.01). The total equivalent value of the NEPHSP was strongly positively correlated with the annual number of service cases (*r* = 0.944, *P* < 0.01), but showed no significant correlation with the unit equivalent value.

**Table 4:** Cost Estimation of the NEPHSP in sample regions

Project	Eastern	Southern	Western	Northern	Central	Guagnxi
Total cost of operational activity(CNY)	189,910,629.23	74,407,942.38	68,632,173.76	69,995,025.99	89,872,983.57	492,818,754.93
Total annual equivalent value of institutional operational activities(Unit)	9,918,708.96	8,596,446.46	3,130,390.91	2,535,840.92	3,200,685.28	27,382,072.53
Cost of one standard service equivalent(CNY)	19.15	8.66	21.92	27.60	28.08	18.00
Total annual equivalent value of the NEPHSP (Unit)	3,910,164.47	2,931,318.24	1,121,255.62	1,285,664.27	1,200,534.55	10,448,937.15
Proportion of the NEPHSP Equivalent Value to Total (%)	39.42	34.10	35.82	50.70	37.51	38.16
Estimated Total Cost of the NEPHSP (CNY)	74,879,649.60	25,385,215.96	24,577,923.19	35,484,333.85	33,711,010.16	188,080,868.70
Number of registered residents served(people)	586,297	364,804	180,562	301,082	262,360	1,695,105
Per capita estimated cost(CNY)	127.72	69.59	136.12	117.86	128.49	110.96

### Disparity analysis of total equivalent value contributions among the NEPHSP

A variance analysis of the proportions of total equivalent values among the 13 NEPHSP categories in the revealed significant differences across sample regions (*F* = 51.061, *P* < 0.05). Health education project had the highest average proportion (26.52%), followed by elderly health management (26.35%) and establishment of resident health record (25.49%). Together, these three projects accounted for a total proportion of 78.36%. Including preventive inoculation (6.20%) and chronic disease management (hypertension) (6.05%), the top five categories comprised 90.61% of the total equivalent value (see Table 5).

**Table 5:** Variance analysis of proportions of total equivalent values for the NEPHSP

13 categories of the NEPHSP	*n*	Mean	SD	F	*P*
Health education	5	26.52	8.90	51.061	0.000**
Elderly health management	5	26.35	3.91		
Establishment of resident health record	5	25.49	4.98		
Preventive inoculation	5	6.20	4.18		
Chronic disease management (hypertension)	5	6.05	2.37		
Traditional Chinese medicine health management	5	2.99	0.54		
Children health management	5	2.76	1.61		
Chronic disease management (type 2 diabetes)	5	1.44	0.66		
Maternal health management	5	1.30	0.23		
Severe mental disorder management	5	0.35	0.05		
Supervision and Co-Management of Health and Family Planning	5	0.32	0.18		
Tuberculosis patient management	5	0.13	0.08		
Reporting and management of infectious diseases and public health emergencies	5	0.10	0.06		

*P < 0.05, **P < 0.01

### Disparity analysis of unit equivalent values for the NEPHSP among counties

Variance analysis of the unit equivalent values for the 13 NEPHSP categories in the sample regions indicated significant inter-county differences for two services: supervision and co-management of health and family planning (*F* = 6.346, *P* < 0.01) and establishment of resident health record (*F* = 2.836, *P* < 0.05). No significant inter-county differences were observed for the remaining service items.

Among all categories, the three highest average unit equivalent values were: health education (58.24), elderly health management (18.60) and preventive inoculation (12.23). The lowest were severe mental disorder management (4.51), traditional Chinese medicine health management (3.66), and establishment of resident health record (2.31)(see Table 6).

**Table 6:** Variance analysis of unit equivalent values for the NEPHSP across counties

13 categories of the NEPHSP	*n*	Mean	SD	F	*P*
Health education	5	58.24	35.02	1.337	0.249
Elderly health management	5	18.60	17.25	1.239	0.300
Preventive inoculation	5	12.23	10.88	1.062	0.411
Maternal health management	5	9.19	8.02	1.613	0.145
Supervision and co-management of health and family planning	5	8.07	6.36	6.346	0.000**
Tuberculosis patient management	5	6.88	8.06	0.704	0.702
Chronic disease management (hypertension)	5	6.58	8.33	1.178	0.335
Chronic disease management (type 2 diabetes)	5	6.10	7.97	1.365	0.236
Reporting and management of infectious diseases and public health emergencies	5	6.02	7.24	0.913	0.524
Children health management	5	5.93	6.71	1.075	0.402
Severe mental disorder management	5	4.51	4.21	0.342	0.955
Traditional Chinese medicine health management	5	3.66	3.62	1.392	0.224
Establishment of resident health record	5	2.31	1.85	2.836	0.011*

*P < 0.05, **P < 0.01

## Discussion

Comparative Analysis of Per Capita Costs for the NEPHSP Under Different Scenarios showed the following trend: per capita actual subsidy (CNY 57.78) < per capita actual expenditure (CNY 60.35) < per capita allocated subsidy (CNY 74) < per capita estimated cost (CNY 110.96). The relatively low per capita actual subsidy reflects its limitation to the annual funds allocated, whereas actual expenditures also drew upon previous years’ carryover balances. In 2020, sampled institutions relied not only on current-year funding but also on prior-year serves to cover project costs, resulting in per capita expenditures exceeding per capita subsidies. Field investigations revealed that fund carryovers resulted from delayed disbursements by county-level finance departments. Financial officers noted that central and provincial government subsidies were the primary and most timely sources for the NEPHSP. However, local fiscal constraints let to lump-sum payment late in the year, restricting effective fund utilization and resulting in carryovers. Such delayed undermine annual targets and population health outcomes, highlighting the NEPHSP’s heavy dependence on government financing. To address these challenges, financially constrained counties should consider engaging professional health management companies through a collaborative approach between finance and health departments, via “service first, payment later” model under contractual agreements which can relieve local financial pressures. Furthermore, leveraging the company’s strengths in health education, hypertension management, and chronic disease patient care ensures that residents in the jurisdiction continue to have access to high-quality basic public health services.

The gap between per capita actual and allocated subsidies indicates a discrepancy bewteen received and intended disbursement. According to China’s Administrative Measures for Subsidy Funds for the NEPHSP, funding allocations are based on population figures from two years prior, resulting in a mismatch when populations decline over time. Specifically, the 2020 funding was calculated based on the 2018 population data. Under this mechanism, if the 2018 service population in reached or exceeded 1.3235 million (calculated as CNY 97,939,575.40 ÷ CNY 74/person), a funding shortfall would occur. Data from Guangxi Statistical Yearbook 2021 confirmed that rural populations have steadily declined since 2002, with figures of 23.83 million 2018, 23.43 million in 2019, and 22.98 million in 2020. It is therefore reasonable to inferr that the 2018 service population was not only higher than the 2020 (1.6961 million) but also well above the 1.3235 million threshold, suggesting that the actual government funding may have been insufficient relative to the intended disbursement.

A comparative analysis of the number of registered residents served and the actual subsidy amounts across regions further confirms the existence of a funding gap. Theoretically, if subsidies were fully aligned with population size, funding would scale proportionally across regions. However, the southern region served more registered residents (364,800) than the central region (262,360), yet received less funding (CNY 14,657,212.10 vs. CNY 18,263,336.54). This discrepancy indicates that the per capita actual subsidy in the southern region was lower than in the central region, indirectly confirming a funding gap. In addition, the study identifiy a weak positive correlation between actual subsidy disbursement and county-level GDP, suggesting that higher levels of socioeconomic development are associated with greater capacity for protective fiscal spending. As a result, the southern region faces a dual challenge. First, limited economic development restricts fiscal capacity, hindering adequate investment in basic public health services. Second, the large service population increases the pressure to ensure health equity, thereby exacerbating supply-demand imbalances in public health services. To address this issue, three policy recommendations are proposed: First, increase fiscal transfers from central and provincial governments to regions with lower per capita actual subsidies to order to promote equitable access to the NEPHSP. Second, strengthen local economic development in underdeveloped areas to enhance the capacity of municipal and county-level government to provide matching fund and increase protective publich health expenditures. Third, diversify funding sources through the establishment of public-private partnership funds. For instance, offering tax incentives and brand promotion opportunities could encourage enterprises to support targeted public health initiatives through donation. Priority should be given to allocating these funds to regions with low per capita actual subsidies, particularly those with large service populations and less-develop economies, to promptly address funding shortfalls.

The significantly higher per capita estimated cost, relative to other indicators, is attributed to the hight high equivalent value NEPHSP activities within overall operations of township health centers. The study found that the NEPHSP activities accounted for 38.16% of the total equivalent value across all sampled institutions, with regional proportions ranging from 34.10 to 50.70%. By contrast, the share of actual subsidy funding was only 16.47% (range from 14.21 to 19.44%), while actual expenditures accounted for 20.76% (range: 17.83–26.42%). These discrepancies indicate a misalignment between human resource input and the corresponding level of financial support. In other words, frontline healthcare workers devote substantial labor to the NEPHSP related tasks, but the financial compensation remain insufficient. Field investigations confirmed the prevalence of a practice known as “supporting the NEPHSP through primary medical revenue”. For instance, when clinical doctors conduct home visits as part of family doctor teams work under NEPHSP is recorded as primary medical service expenditure rather then as a cost to the public health program. Similarly, when NEPHSP funding is in shortages, shared administrative expenses—such as business travel and office supplies—are often covered by primary medical accounts. From a policy perspective, existing financial regulations explicitly prohibit the reallocation of the NEPHSP funds for non-designated services, however, there are no corresponding restrictions on using primary medical revenues to cover the NEPHSP expenditures. While the practice of the “supporting the NEPHSP through primary medical revenue” can temporarily ease financial pressures and enable township health centers meet their performance targets, thus ensuring equal and free access to various health management services for rural residents. it may also undermine the long-term sustainability of these institutions and reduce the motivation of clinical staff. The study further found that, due to the NEPHSP are fully funded by the government, financial and clinical personnel in township health centers generally exhibit weak awareness of cost contro. Interviewees frequently reported insufficient funding for service delivery, yet lacked clarity on which specific service incurred the greatest costs or required the most labor. To address this issue, it is recommended that cost-control modules be integrated and emphasized with the annual training sessions for the NEPHSP. Improving awareness of cost-efficiency and adopting evidence-based cost accounting practices would enable township health centers to allocation limited healthcare resources more effectively and enhance service delivery outcomes.

The findings reveal a weak positive correlation between per capita estimated cost and the proportion of total equivalent value tothe NEPHSP, along with notable variations in the total equivalent value ratios across different services. This suggests that services with higher total equivalent value ratios substantially contributing to elevated per capita costs. Additionally, a strong positive correlation exists between the total equivalent value of each service and its corresponding annual workload. For instance, five services categories that comprise 90.61% of the total equivalent value rank among the top five in annual workload volume. Establishing resident health record and conducting health education rank first and second, respectively, as both target the entire population. Preventive inoculation ranks third due to its inclusion of eight vaccines types including hepatitis B vaccine and BCG vaccine, which resulting in a high volume of routine vaccinations among children. Elderly health management comes fourth. With China’s ongoing urbanization and aging demographic, the proportion of elderly people in rural areas is rising intensifying demand for elderly health management services. Chronic disease management (hypertension) ranks fifth, highlighting the large number of hypertensive patients as a key demographic requiring targeted intervention. Therefore, it is recommended that both the Health Commission, as the regulatory authority and township health centers, as service providers, prioritize high workload services to address the primary health needs of rural populations, while also ensuring foundational support for the eight service categories comprising the remaining 10% of the total equivalent value to enhance fairness and equity in the provision of basic public health services. Furthermore, attention should be paid to potential shifts in health management needs driven by demographic changes. As China’s population continue to age, rising demand for elderly health management and chronic disease management, particularly for hypertension and diabetes will place increasing strain on service delivery capacity of of township health centers.

Among the 13 categories of basic public health service programs, the unit equivalent values of 11 categories exhibited no significant inter-county variation, suggesting a relatively high level of standardization in the implementation of these services across counties. However, notable regional variations were observed in the unit equivalent values for two specific services: establishing resident health record and the supervision and co-management of health and family planning. Field investigations indicated that discrepancies in the unit equivalent values for establishing health records were primarily related to healthcare workers’ varying leve of proficiency in using the information system, their communication skills with residents, and their capacity to update records promptly. To address these challenges,, it is recommended to strengthen pre-service training programs amied at improving both communication and digital literacy competencies among healthcare staff. additionally, optimizing the health record management system through features such as automatic data linkage and validation is suggested to enhance usability and operational efficiency. The observed variation in the unit equivalent value for the supervision and co-management of health and family planning was linked to regional geographical characteristics. In Guangxi, where population distribution is characterized by “large-scale intermingling with small-scale clustering” pattern and rural area are predominantly mountainous, conducting supervision activities in remote regions demands greater personnel and time investment. This highlights the potential impact of geographic factors on the efficiency of resource allocation and complexity of implementation primary-level public health services. Relying solely on population size as the basis for resource distribution may lead to reduced service accessibility and disparities in service quality. To mitigate these challenges, the introduction of a geographic weighting factor into resource allocation models is recommended, ensuring preferential support for remote and transportation-disadvantaged areas. Furthermore, the use of mobile technologies such as drones and other portable devices could facilitate remote supervision and reduce labor costs, thereby improving overall service delivery efficiency.

## Conclusion

This study elucidated the interrelationships between actual per capita subsidies, actual expenditures, allocatedsubsidies, and estimated costs. Key findings include: (1) Discrepancies between actual financial subsidies and planned allocations in certain counties, aggravated by local fiscal constraints and delays fund disbursements, hindered the achievement of annual service targets and compromised the equitable implementation of the NEPHSP; (2) In several counties, mismatches between population size and fiscal subsidies dual pressures: limited fiscal capacity and substantial service populations, highlighting the urgent need to optimize transfer mechanisms and develop diversified financing strategies; (3) Reliance on primary healthcare revenues to fund NEPHSP implementation revealed systemic weaknesses in fiscal safeguards systems in some areas. The total equivalent value analysis identified substantial dispariteis in workload across service categories, emphasizing the need for resource allocation strategies that balance responsiveness to demand with equity considerations. Unit equivalent value analysis indicated satisfactory standardization of most NEPHSP services across counties, however, notable regional variations remained in health record establishment and health supervision coordination, driven by disparities in workforce capacity, information system maturity, and geographic constraints.

Based on above findings, several targeted policy recommendations are proposed. First Central and provincial governments should increase fiscal transfer payments to regions with low per capita actual subsidies. Second economically underdeveloped region should prioritize local economic development to enhance their ability to match funds. Third, diversified funding sources should be explored, including public-private partnerships and other innovative financial models to foster collaborative social governance. Finally, policy efforts should focus on strengthening staff capacity building, information systems optimaztion, and the application of geographic weighting factors to improve the equity and efficiency of resource allocation. These measures aim to promote the sustainable and high-quality development of the NEPHSP and ultimately achieve the goal of service equity and equal access.

## Limitations

This study has several limitations that should be acknowledged. First, the number of registered residents served was obtained from township health center records rather than from the official figures used for government budget allocations, as a result, it was not possible to accurately estimate the discrepancy between actual funding received and expected allocation based on official population data. Second, the cost estimation of per capita service costs based on equivalent value method included only labor expenses and secluded other key cost components such as medications, medical consumables, and diagnostic and laboratory services. this limitation may have led to an overestimation the actual per capita estimated cost of delivering basic public health services. Third, the absence of established reference standards for work efficiency across service categories limits the interpretation of unit equivalent value. While these values help analyze regional differences in workload, they do not allow for an assessment of whether service quality meets the program’s required standards (e.g., more comprehensive data collection and meticulous follow-up records may require additional time, and this information could potentially become crucial for health management). Future studies should expand the sample size and incorporate quality assessment indicators to enhance the robustness and generalizability of the findings.

## Data Availability

The data that support the findings of this study are available from [Health Commission of Guangxi of China] but restrictions apply to the availability of these data, which were used under license for the current study, and so are not publicly available. Data are however available from the authors upon reasonable request and with permission of [Health Commission of Guangxi of China].

## Abbreviations

NEPHSP: China’s National Essential Public Health Service Package
Guangxi: Guangxi Zhuang Autonomous Region
WISN: Workload Indicators of Staffing Need
WHO: World Health Organization
